# Dual Machine Learning Framework for Predicting Long-Term Glycemic Change and Prediabetes Risk in Young Taiwanese Men

**DOI:** 10.3390/diagnostics15192507

**Published:** 2025-10-02

**Authors:** Chung-Chi Yang, Sheng-Tang Wu, Ta-Wei Chu, Chi-Hao Liu, Yung-Jen Chuang

**Affiliations:** 1 Division of Cardiovascular Medicine, Taoyuan Armed Forces General Hospital, Taoyuan 325208, Taiwan; t220979@gmail.com; 2 Cardiovascular Division, Tri-Service General Hospital, National Defense Medical University, Taipei 11490, Taiwan; 3 School of Medicine, National Tsing Hua University, Hsinchu 30044, Taiwan; 4 Institute of Bioinformatics and Structural Biology, National Tsing Hua University, Hsinchu 30044, Taiwan; 5 Division of Urology, Department of Surgery, Tri-Service General Hospital, National Defense Medical University, Taipei 11490, Taiwan; doc20283@gmail.com; 6 Division of Urology, Department of Surgery, Kaohsiung Armed Forces General Hospital, Kaohsiung 802301, Taiwan; 7 Department of Obstetrics and Gynecology, Tri-Service General Hospital, National Defense Medical University, Taipei 11490, Taiwan; taweichu@gmail.com; 8 MJ Health Research Foundation, Taipei 114066, Taiwan; 9 Division of Nephrology, Department of Internal Medicine, Kaohsiung Armed Forces General Hospital, Kaohsiung 802301, Taiwan; 10 School of Medicine, National Defense Medical University, Taipei 11490, Taiwan

**Keywords:** change of fasting plasma glucose, Taiwanese, machine learning, prediabetes, young men

## Abstract

**Background:** Early detection of dysglycemia in young adults is important but underexplored. This study aimed to (1) predict long-term changes in fasting plasma glucose (δ-FPG) and (2) classify future prediabetes using complementary machine learning (ML) approaches. **Methods:** We analyzed 6247 Taiwanese men aged 18–35 years (mean follow-up 5.9 years). For δ-FPG (continuous outcome), random forest, stochastic gradient boosting (SGB), eXtreme gradient boosting (XGBoost), and elastic net were compared with multiple linear regression using Symmetric mean absolute percentage error (SMAPE), Root mean squared error (RMSE), Relative absolute error(RAE), and Root relative squared error (RRSE) Sensitivity analyses excluded baseline FPG (FPG_base_). Shapley additive explanations(SHAP) values provided interpretability, and stability was assessed across 10 repeated train–test cycles with confidence intervals. For prediabetes (binary outcome), an XGBoost classifier was trained on top predictors, with class imbalance corrected by SMOTE-Tomek. Calibration and decision-curve analysis (DCA) were also performed. **Results:** ML models consistently outperformed regression on all error metrics. FPG_base_ was the dominant predictor in full models (100% importance). Without FPG_base_, key predictors included body fat, white blood cell count, age, thyroid-stimulating hormone, triglycerides, and low-density lipoprotein cholesterol. The prediabetes classifier achieved accuracy 0.788, precision 0.791, sensitivity 0.995, ROC-AUC 0.667, and PR-AUC 0.873. At a high-sensitivity threshold (0.2892), sensitivity reached 99.53% (specificity 47.46%); at a balanced threshold (0.5683), sensitivity was 88.69% and specificity was 90.61%. Calibration was acceptable (Brier 0.1754), and DCA indicated clinical utility. **Conclusions:** FPG_base_ is the strongest predictor of glycemic change, but adiposity, inflammation, thyroid status, and lipids remain informative. A dual interpretable ML framework offers clinically actionable tools for screening and risk stratification in young men.

## 1. Introduction

The global prevalence of type 2 diabetes (T2D) continues to rise, with an estimated 529–537 million adults affected in 2021 and projections reaching 1.31 billion by 2050 [1,2]. In Taiwan, NAHSIT 2013–2016 data show that nearly one-third of adults have impaired fasting glucose [3], yet only 4.1% of T2D patients meet the American Diabetes Association’s goals for glycated hemoglobin, blood pressure, and cholesterol [4].

The age of diabetes onset has been decreasing [5]. Because longer disease duration is strongly correlated with complications, younger patients are expected to experience more complications over their lifetime [6]. Accordingly, the American Diabetes Association recommends diabetes screening from age 35 [7].

Statistical and machine learning approaches have been used to predict diabetes or prediabetes in Chinese, Korean, and other cohorts [8,9,10,11,12], but most studies were cross-sectional or focused on older populations [8,9,10], with few targeting young adults [12]. In addition, interpretability methods such as Shapley additive explanations (SHAP) are rarely applied [13,14], and prior models typically addressed a single outcome (continuous change or binary classification). Table 1 summarizes these studies for comparison.

In contrast, our work leveraged a large, longitudinal cohort of young Taiwanese men with nearly six years of follow-up, employed a dual machine learning (ML) framework (continuous fasting plasma glucose (δ-FPG) and binary prediabetes), integrated SHAP for interpretability, and ensured reproducibility through repeated runs and confidence intervals. These strengths make our model more clinically actionable for early prediabetes risk stratification. Recent reviews further endorse SHAP and other explainable AI (XAI) tools for transparent clinical decision support [15,16].

This interpretability is particularly valuable in binary ML classification models, which predict categorical outcomes such as the presence or absence of prediabetes. Unlike continuous models that track gradual metabolic changes, binary methods align directly with diagnostic thresholds used in clinical practice [17,18,19], making them particularly suited for screening programs and early intervention planning. When combined with SHAP, binary models not only deliver high predictive accuracy but also allow clear identification of the most influential factors driving a classification decision. This dual capacity—accurate prediction and transparent reasoning—makes binary ML models an essential complement to continuous prediction frameworks, ensuring both statistical robustness and clinical applicability.

In the present study, we enrolled 6247 young Taiwanese men followed up for 5.9 years. By using four different ML methods, our objectives were as follows:

1. Compare the accuracy of multiple logistic regression (MLR) and ML approaches.

2. Identify the most important risk factors for δ-FPG, defined as FPG at the end of follow-up minus FPG at baseline (FPG_base_).

3. Use SHAP to further examine the impact and direction of each factor.

In addition to modeling δ-FPG as a continuous variable, we also aimed to develop and evaluate a categorical (binary) ML model to classify individuals as normal or prediabetes based on demographic, biochemical, and lifestyle factors, thereby aligning predictive modeling with clinically relevant diagnostic thresholds.

## 2. Materials and Methods

### 2.1. Participant and Study Design

The methods used in this study were described in our previous publication [11]. Data were obtained from the Taiwan MJ cohort, an ongoing prospective cohort based on health examinations conducted by the MJ Health Screening Centers in Taiwan [20]. These examinations include more than 100 biological indicators, such as anthropometric measurements, blood tests, and imaging studies. Each participant also completed a self-administered questionnaire covering personal and family medical history, current health status, lifestyle, physical activity, sleep habits, and dietary patterns [20].

This study was a secondary data analysis. At the time of health examinations, participants provided general consent for future anonymous research use. The database is maintained by the MJ Health Research Foundation. All or part of the data used in this study were authorized and provided by the MJ Health Research Foundation (Authorization Code: MJHRF2023007A). The interpretations and conclusions presented herein do not necessarily represent the views of the foundation. For further details, please refer to the foundation’s annual technical reports [21].

The study protocol was approved by Institutional Review Board of the Kaohsiung Armed Forces General Hospital (IRB No.: KAFGHIRB 112-006, date of approval 21 June 2023). Since there was no sample collection from the patients, a short review of the IRB was approved, and no consent form was needed. A comprehensive list of variables used in the analysis is provided in Table 2, including demographic, biochemical, and lifestyle factors. All variables are reported with their original units (e.g., %, ×10^3^/μL, mg/dL). Clinically relevant transformations—such as log-transformations for triglycerides (TG), γ-glutamyl transpeptidase (γ-GT), and alkaline phosphatase (ALP), as well as derived ratios (TG/high density lipoprotein cholesterol (HDL-C), low density lipoprotein cholesterol (LDL-C)/HDL-C, waist-to-hip ratio)—were applied as part of feature engineering and are consistent with metabolic risk assessment guidelines.

Initially, 47,666 men were enrolled. For this study, we specifically selected participants aged 18–35 years, as described in the Introduction. The exclusion criteria were as follows:

1. Age < 18 and >35 years old.
2. Use of medications known to affect blood pressure, blood glucose, or blood lipids.
3. Abnormal plasma glucose level at baseline.

**Figure 1:**
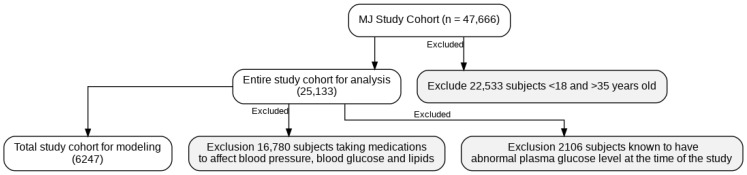
The scheme of participant selection.

After excluding subjects who did not fit our inclusion criteria, 6247 men were left for further analysis (Figure 1). Methods of how to collect demographic, biochemistry, and lifestyle data were published by our group previously and are not shown here [19].

Table 2 shows the 25 baseline variables. These included participants’ age, body fat (BF), complete blood cell count, biochemistries, thyroid stimulating hormone (TSH), c-reactive protein (CRP), education level, relationship status, and income level. The drinking area was defined as the multiple of total drinking duration, frequency of drinking, and the percentage of alcohol. Similarly, the smoking area was the multiple of the duration, frequency of smoking, and number of cigarettes. The sport area was the multiple of duration, frequency, and type of exercise. All the aforementioned parameters were the independent variables, and the dependent variable was δ-FPG after an average of 5.9 years follow-up.

Two models were built in the present study. From our preliminary evaluation, Model 1 included all 25 variables. Our results showed that FPG_base_ had 100% importance compared to the second important factor (BF, 17.6%). In order to further evaluate the hidden interactions between these factors, Model 2 was built by removing the FPG_base_.

### 2.2. Traditional Statistical Method

MLR was used, with δ-FPG as the dependent variable and demographic, biochemical, and lifestyle variables as independent predictors. Further technical details were provided in our previous publication [12].

### 2.3. Machine Learning Methods

We employed two complementary ML frameworks to predict prediabetes risk and identify key predictors from demographic, biochemical, and lifestyle data. The first modeled the outcome as a continuous variable—δ-FPG—while the second modeled a binary outcome (normal vs. prediabetes). This dual design allowed us to capture both fine-grained metabolic variation and clinically actionable thresholds.

#### 2.3.1. Continuous Outcome Prediction (δ-FPG)

**Figure 2:**
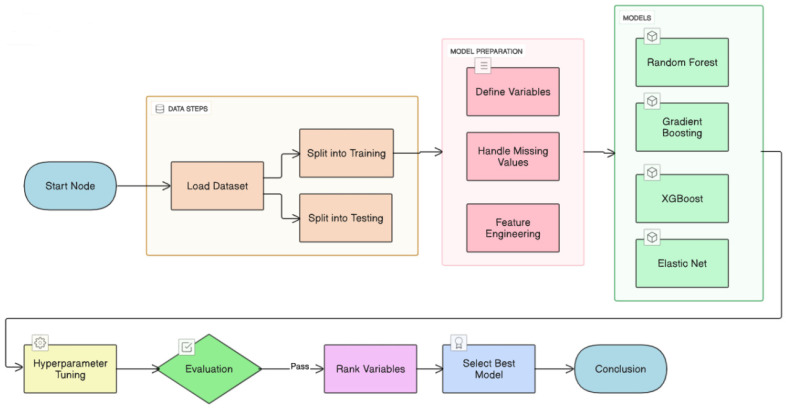
Proposed machine learning prediction scheme.

Four ML algorithms—random forest (RF) [22], stochastic gradient boosting (SGB) [23], eXtreme gradient boosting (XGBoost) [24], and elastic net (EN) [25]—were trained to predict δ-FPG (Figure 2). Data were split 80/20 into training and testing sets. Hyperparameters were tuned via 10-fold cross-validation with grid search. Performance was assessed with symmetric mean absolute percentage error (SMAPE), relative absolute error (RAE), root relative squared error (RRSE), and root mean squared error (RMSE) (Table 3).

To ensure robustness, the training/testing procedure was repeated 10 times with different random seeds. The mean and 95% confidence intervals (CIs) of performance metrics across these runs demonstrate model stability and reproducibility. Feature importance scores were averaged across all runs and models. Feature importance distributions were examined using boxplots of SHAP-derived importance, generated from 20 repeated training–testing cycles with different random seeds. Narrow boxplots indicate stable contributions across runs, whereas wider distributions highlight features with unstable or context-dependent importance. This procedure has been recommended for evaluating the reliability of explanation methods in machine learning [26].

A planned sensitivity analysis removed FPG_base_ due to its dominant influence (100% importance in initial models). The same modeling procedures were applied to Model 2 (without FPG_base_) to identify alternative predictors. Feature importance for both Model 1 and Model 2 are presented in Section 3.3.

Model interpretability for the FPG_base_-excluded model (Model 2) was further explored using SHAP applied to the random forest algorithm. The SHAP bee swarm plot visualizes the direction and magnitude of each feature’s impact on individual predictions. The mean absolute SHAP values provide a global ranking of feature importance, while the net SHAP values indicate whether features exert predominantly positive or negative effects overall. Dependence plots for key features (e.g., follow-up interval and baseline FPG) are also shown in Section 3.3, illustrating how feature effects are modulated by other variables.

Residual diagnostics for the continuous models, including quantile–quantile plots and residuals vs. fitted values, were used to validate model assumptions.

#### 2.3.2. Binary Outcome Prediction (Normal vs. Prediabetes)

For classification, we developed an XGBoost-based pipeline [24] using a 70/30 derivation/validation split. Feature engineering included clinically relevant ratios (TG/HDL-C, LDL-C/HDL-C, waist-to-hip ratio) and log transformations (TG, γ-GT, ALP). Missing data were imputed using median values [27]. Class imbalance was addressed with SMOTE-Tomek [28,29]. We also considered recent evidence and reviews on imbalance remedies to mitigate over-optimism and miscalibration risks [30,31]. Feature selection used permutation importance from a baseline XGBoost model, retaining the top 20 predictors. Hyperparameters were tuned with Optuna [32] in threefold stratified CV. Final model performance was evaluated on the validation set using accuracy, precision, recall, specificity, F1 score, ROC-AUC, and PR-AUC. Model interpretability was assessed with SHAP [13]. The confusion matrix at a threshold of 0.50 is also shown. Given class imbalance, precision–recall curves were prioritized for conveying retrieval performance [33]. All preprocessing steps—including median imputation, SMOTE-Tomek resampling, feature engineering, and feature selection—were applied independently within each training fold during cross-validation to prevent data leakage. Hyperparameter tuning was performed using Optuna with threefold stratified CV, 100 trials, early stopping after 20 rounds, and a fixed random seed (42) for reproducibility.

To prevent data leakage, all preprocessing steps—including missing value imputation, feature engineering, SMOTE-Tomek resampling, and feature selection—were applied independently within each training fold during cross-validation. Specifically, the median for imputation was computed solely from the training fold and applied to both training and validation subsets. Similarly, SMOTE-Tomek was applied only to the training fold, without incorporating any validation data. Feature selection based on permutation importance was performed using a model trained on the training fold only. Model calibration was assessed using the Brier score and a reliability (calibration) curve, where predicted probabilities were compared with observed outcomes across 10 bins of predicted risk, providing a visual assessment of calibration. Brier score is defined as

$$Brier\;Score=\frac{1}{n}\sum_{i=1}^{n}{\left({\hat{p}}_{i}-yi\right)}^{2}$$

where ${\hat{p}}_{i}$ is the predicted probability of prediabetes for the i-th individual, yi is the observed outcome (1 if prediabetic, 0 otherwise), and *n* is the number of individuals in the validation set. A lower Brier score indicates better calibration, with 0 representing perfect calibration. These choices align with recent BMJ guidance on evaluating clinical prediction models, covering discrimination, calibration, and external validation [34,35].

To evaluate the clinical utility of the model across different decision contexts, we assessed two operating thresholds: (1) a high-sensitivity threshold (0.2892) optimized to minimize false negatives, which is appropriate for screening settings, and (2) a balanced threshold (0.5683) identified using Youden’s J statistic, which maximizes the sum of sensitivity and specificity. The optimal threshold was determined by maximizing Youden’s J = (sensitivity + specificity − 1). Decision-curve analysis (DCA) was performed to quantify the net benefit of the model across a range of threshold probabilities, comparing it against strategies of treating all or none. DCA was performed to evaluate the clinical utility of the binary classifier by quantifying the net benefit of using the model to guide clinical decisions across a range of threshold probabilities [36,37]. The net benefit was calculated as the difference between the proportion of true positives and the weighted proportion of false positives, where the weight is the threshold probability divided by (1-threshold probability). This allowed the comparison of the model’s performance against two extreme strategies: treating all patients or treating none. DCA was implemented using the predicted probabilities from the validation set and the true outcome labels, across threshold probabilities ranging from 0.01 to 0.99.

#### 2.3.3. Rationale for Combined Framework

The continuous framework offers precise measurement of glycemic change, while the binary framework aligns with diagnostic thresholds for prediabetes. Presenting both ensures statistical rigor and clinical relevance.

#### 2.3.4. Confidence Interval Estimation

To quantify the uncertainty around model performance, we calculated 95% CIs for each evaluation metric (SMAPE, RMSE, RAE, RRSE) based on the distribution of scores obtained from 10 repeated training–testing cycles for each model. For each metric, the mean and standard error (SE) were computed, and the CIs was estimated as:

$$CIs\;=\bar{x}\pm \;t{\alpha /}_{2},\;n-1\;\times \;SE$$

CIs = $\bar{x}$ ± tα/2, n − 1 × SE, where $\bar{x}$ is the sample mean of the metric, tα/_2_; n − 1 is the critical value from the Student’s t-distribution with n − 1 degrees of freedom [38]; and SE is the standard error of the mean. This approach follows established statistical practice for small-sample inference [39] and is applicable when the sampling distribution of the metric is approximately normal [40].

#### 2.3.5. Sensitivity and Ablation Analysis

To assess model robustness and the concentration of predictive signal, we performed an ablation analysis by removing the top 3 most important features from the binary XGBoost model. The resulting drop in ROC-AUC is reported in Supplementary Table S1, illustrating the model’s dependence on key predictors.

#### 2.3.6. Multicollinearity Assessment

To ensure feature independence and model stability, we computed the variance inflation factor (VIF) for all predictors. Results are presented in Supplementary Table S2, with all VIF values below 10, indicating acceptable levels of multicollinearity. The feature correlation heatmap is shown in Supplementary Figure S1. These diagnostic tools help ensure that observed feature importance is not an artifact of highly correlated variables, which can distort model interpretability in biomedical datasets [41].

#### 2.3.7. Reproducibility Artifact

To enhance transparency and reproducibility, we provided a minimal, self-contained Python v3.10.12 script that generated synthetic data mimicking the structure of our cohort (n = 100 young Taiwanese men with features including age, body fat, WBC, TSH, TG, LDL-C, and δ-FPG) and executed a simplified version of our modeling pipeline (Supplementary Figure S2). The script trained an XGBoost Regressor to predict δ-FPG, and it computed SHAP values for interpretability and RMSE as a performance metric. The synthetic data were generated using biologically plausible distributions and correlations, ensuring the code demonstrated the core workflow without requiring access to the real, privacy-sensitive MJ Health data. This artifact is available from the corresponding author upon reasonable request and fulfills the journal’s reproducibility guidelines.

## 3. Results

### 3.1. Demographic Characteristics

Our analysis included 6247 young Taiwanese men aged 18–35 years at baseline, with a mean follow-up duration of 5.9 years (Figure 1). The selection scheme and exclusion criteria are detailed in Figure 1. Baseline demographic, biochemical, and lifestyle characteristics of the full cohort are summarized in Table 2.

To contextualize our work, Table 1 compares our study design, population, methods, and findings with four recent studies predicting prediabetes or diabetes risk. Our study is distinguished by its focus on young men, prospective design, dual ML framework (continuous δ-FPG + binary classification), application of SHAP interpretability, and rigorous assessment of reproducibility through repeated runs and CIs.

Participants were stratified by glycemic outcome at follow-up: Group 1 (n = 2789) developed prediabetes, while Group 2 (n = 3458) remained normoglycemic. Significant differences between groups are shown in Table 4. Compared to Group 2, Group 1 participants were older (28.4 vs. 27.3 years, *p* < 0.001) and had higher body fat (22.1 vs. 20.8 mg/dL, *p* < 0.001), higher white blood cell count (6.3 vs. 6.2 × 10^3^/μL, *p* < 0.001), higher baseline FPG (93.3 vs. 91.0 mg/dL, *p* < 0.001), higher triglycerides (105.8 vs. 96.0 mg/dL, *p* < 0.001), and higher LDL-C (115.0 vs. 110.6 mg/dL, *p* < 0.001). They also had shorter follow-up duration (5.6 vs. 6.1 years, *p* < 0.001) and lower HDL-C (48.4 vs. 50.0 mg/dL, *p* < 0.001). Marital status (fewer single men in prediabetes group) and income level (fewer unemployed in prediabetes group) also differed significantly.

### 3.2. Continuous Outcome Prediction (δ-FPG)

We first evaluated the ability of four machine learning models: RF, SGB, XGBoost, and EN, to predict the continuous outcome δ-FPG. Performance metrics (SMAPE, RAE, RRSE, RMSE) and their definitions are provided in Table 5.

Model 1 (all predictors included): All ML models outperformed MLR. Elastic net achieved the lowest RMSE (6.4092) and RRSE (0.9068), followed closely by random forest (6.4133, 0.9074) and XGBoost (6.4329, 0.9102) (Table 5).

Model 2 (FPG_base_ excluded): As expected, performance declined slightly across all models due to removal of the strongest predictor. EN performed best (RMSE: 6.8985, RRSE: 0.9827), followed by SGB (RMSE: 6.8916, RRSE: 0.9817) and RF (RMSE: 6.9175, RRSE: 0.9854) (Table 5).

To assess reproducibility, we repeated the training/testing procedure 10 times. Table 6 presents the mean and 95% confidence intervals for each metric. The intervals were extremely narrow (often identical upper and lower bounds), indicating high stability and reproducibility of model performance across runs. For example, RF RMSE was consistently mean = 6.471158, SE = 0.00179, 95% CI = 6.467109–6.475208.

**Figure 3:**
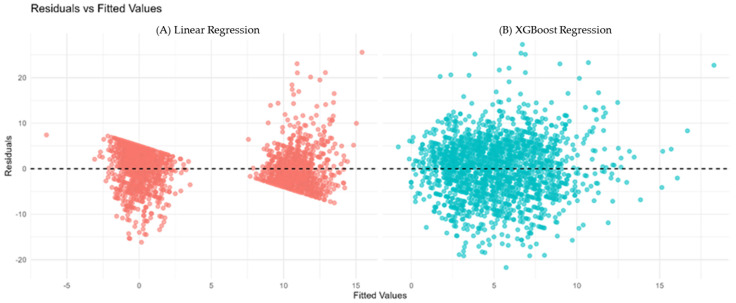
(**A**) Residuals vs. fitted values for Linear Regression (red). (**B**) Residuals vs. fitted values for XGBoost Regression (cyan). The horizontal dashed line at zero indicates no residual bias. Patterns or funnel shapes may suggest heteroscedasticity or model misspecification.

Residual diagnostics for the continuous models, including quantile–quantile plots and residuals vs. fitted values, are presented in Figure 3, confirming reasonable adherence to model assumptions.

### 3.3. Feature Importance and Model Interpretability (Continuous Models)

**Figure 4:**
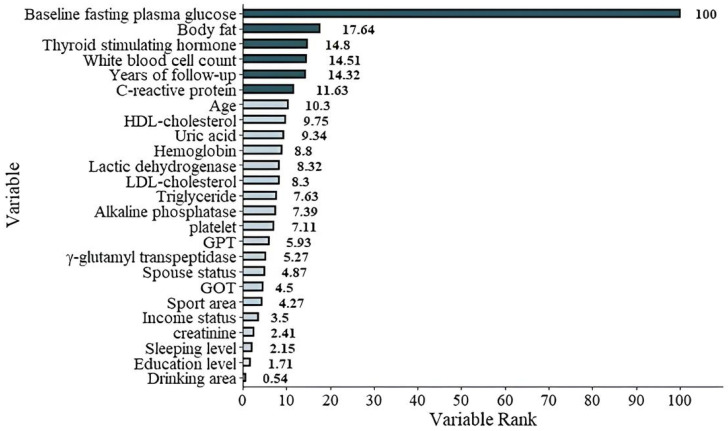
The graphic demonstration of the importance percentage in Model 1 (with FPG_base_ included). The darker green bars show the most important six features of the study.

In the full model (Model 1), which included all baseline predictors, FPG_base_ had the maximum possible importance score of 100% across all four ML methods (Table 7, Figure 4). This overwhelming dominance of FPG_base_ meant that its predictive contribution far exceeded all other variables, with the second-ranked feature, BF, having a mean importance of only 17.6%. Such a strong single-variable effect can mask the influence of other clinically relevant predictors.

**Figure 5:**
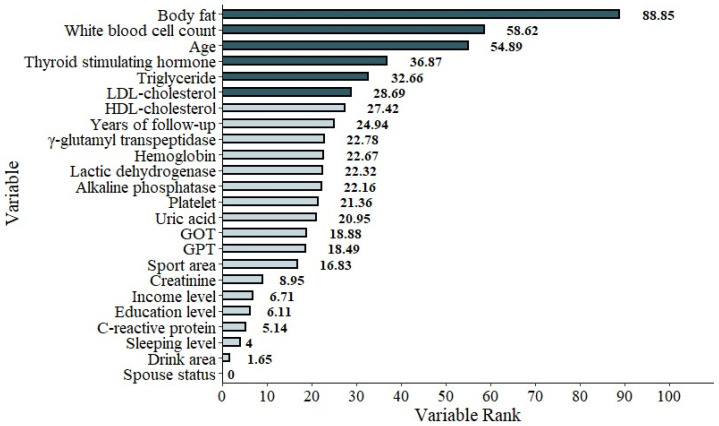
The graphic demonstration of the importance percentage in Model 2 (excluding FPG_base_). The darker green bars show the most important six features of the study.

To address this, we developed a second model (Model 2) with FPG_base_ removed from the predictor set. In this model, BF emerged as the top predictor (mean importance: 88.85%), followed by white blood cell count (WBC, 58.62%), age (54.89%), thyroid-stimulating hormone (TSH, 36.87%), triglycerides (TG, 32.66%), and LDL-C (28.69%) (Table 8, Figure 5). The redistribution of importance scores revealed a broader set of influential features that may otherwise be overlooked when FPG_base_ is included.

Although the exclusion of FPG_base_ led to a slight decline in predictive performance across all ML methods, the results remained stable, suggesting that these secondary predictors retained substantial explanatory value for δ-FPG. This sensitivity analysis highlights that while FPG_base_ is the single strongest predictor, other metabolic, inflammatory, and lipid-related factors also play significant independent roles in determining future changes in fasting plasma glucose.

**Figure 6:**
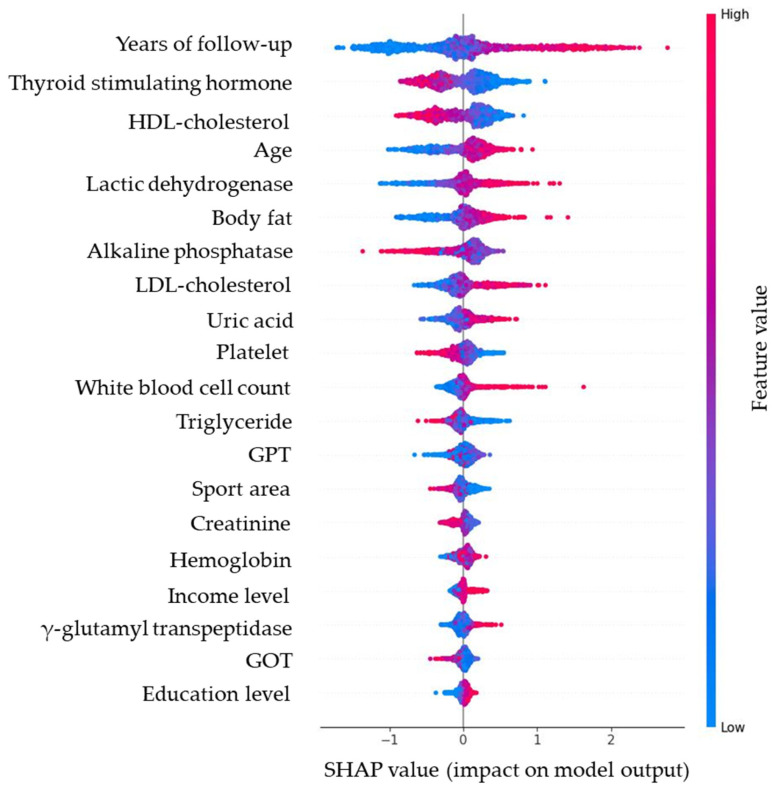
SHAP bee swarm plot of the random forest model, illustrating the distribution and magnitude of predictor effects (red = higher values, blue = lower values). Note: Red dots (higher feature value) push predictions upward (worsening glycemia); blue dots (lower feature value) push predictions downward (improving glycemia). For example, higher body fat consistently increases predicted glucose rise, while higher HDL-C is protective.

Model interpretability was assessed using SHAP applied to the RF model for the FPG_base_-excluded dataset. Figure 6 presents the bee swarm plot, showing the distribution and direction of SHAP values for each predictor. Variables such as years of follow-up, TSH, HDL-C, age, LDH, and BF had the most substantial influence on δ-FPG, with color gradients indicating whether higher feature values were associated with increases (red) or decreases (blue) in predicted δ-FPG.

**Figure 7:**
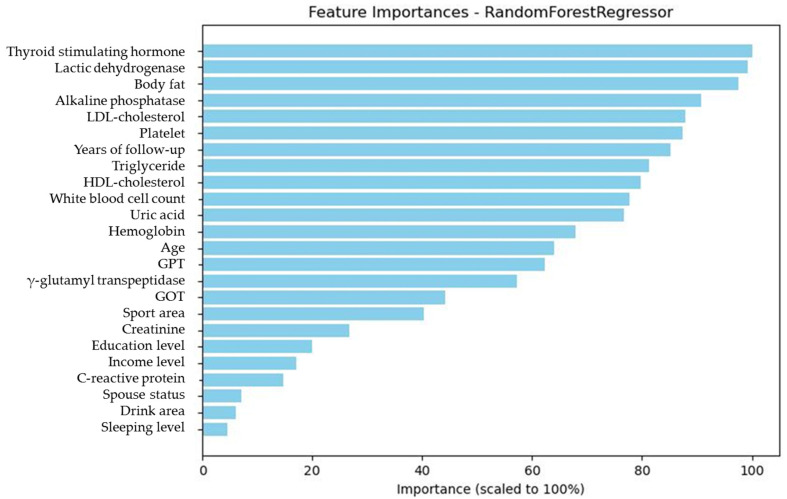
Absolute SHAP value ranking of predictors, showing the magnitude of each variable’s contribution to glycemic trajectory.

Figure 7 shows the mean absolute SHAP values for each feature, ranking them by overall importance. Years of follow-up, TSH, HDL-C, and age remained the top contributors, confirming their consistent predictive role.

**Figure 8:**
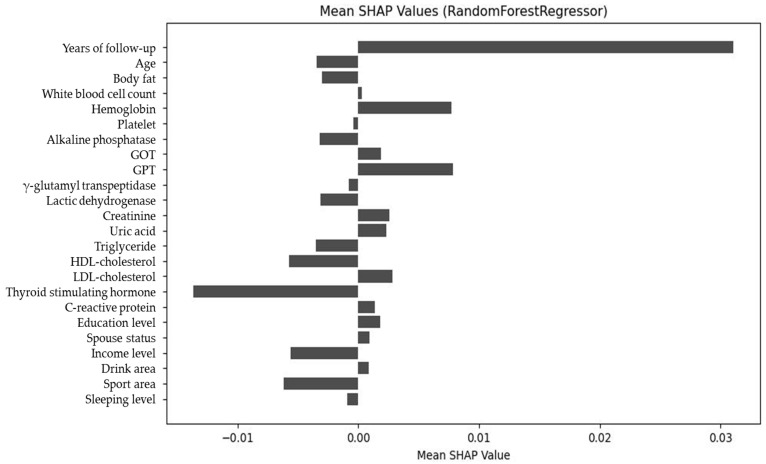
Net SHAP value plot of predictors, demonstrating the overall directional impact of features on glycemic outcomes.

Figure 8 displays the net SHAP values, reflecting the difference between the mean positive and negative contributions for each feature. This plot highlights features whose effects are unidirectional (consistently increasing or decreasing δ-FPG) versus those with bidirectional influence depending on their value. Together, these three visualizations provide a comprehensive understanding of both the magnitude and directionality of each predictor’s impact on model predictions.

**Figure 9:**
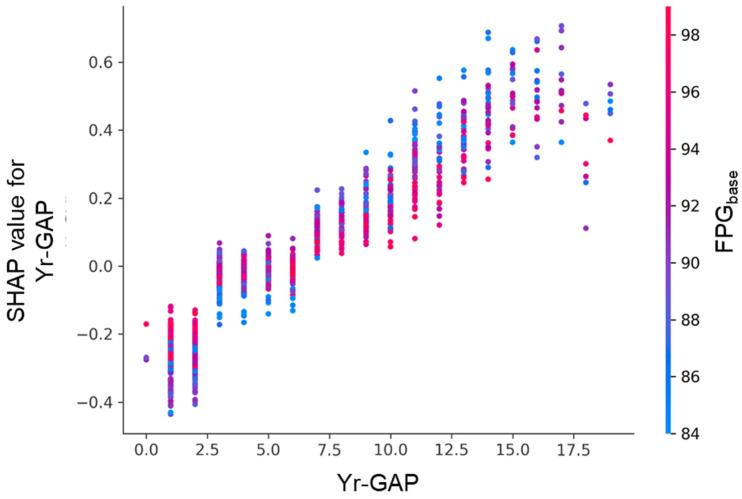
SHAP dependence plot for follow-up interval (Yr-GAP), colored by FPG_base_.

**Figure 10:**
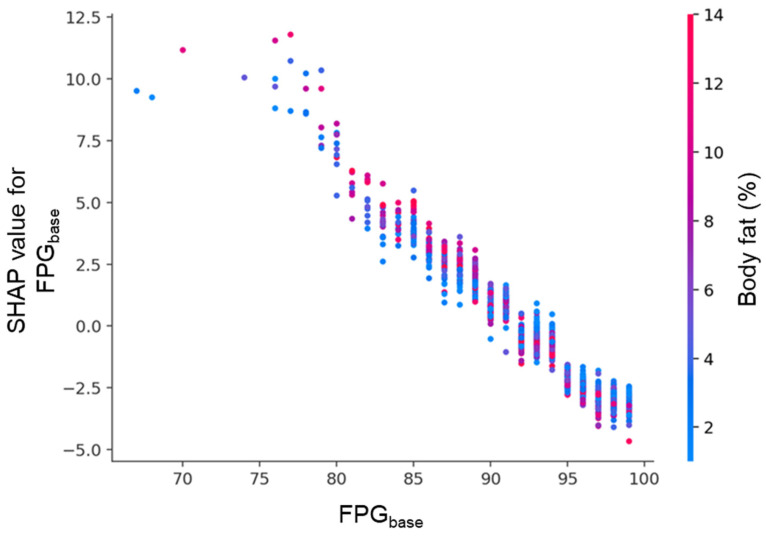
SHAP dependence plot for FPG_base_, colored by body fat percentage. Each point represents an individual participant. The *x*-axis shows FPG_base_, and the *y*-axis indicates its SHAP value. Lower FPG_base_ levels were associated with strong positive SHAP values, while higher levels showed negative contributions. Body fat percentage, represented by the color gradient, further modulated this relationship.

There are two key SHAP dependence plots that illustrate feature interactions. Figure 9 shows the effect of follow-up interval (Yr-GAP) on SHAP values was modulated by FPG_base_. Longer follow-up generally increased risk, but this effect was stronger in individuals with lower FPG_base_. Figure 10 shows the effect of FPG_base_ was modulated by body fat percentage. Lower FPG_base_ was associated with higher risk (positive SHAP), especially in individuals with higher body fat.

Each point represents an individual participant. The *x*-axis shows years between baseline and follow-up examinations (Yr-GAP), and the *y*-axis indicates the corresponding SHAP value. A longer follow-up interval was associated with progressively higher SHAP values, suggesting an increasing contribution to the model prediction. The color gradient denotes FPG_base_, which modulated the effect size.

### 3.4. Binary Outcome Prediction (Prediabetes vs. Normal)

**Figure 11:**
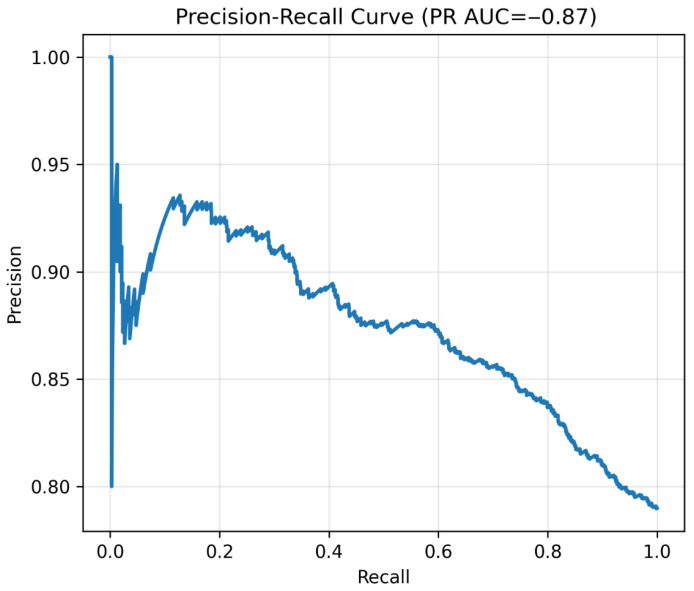
Precision–recall curve. Note: Precision–recall curve of the binary classifier. High recall confirmed excellent sensitivity for prediabetes detection.

**Figure 12:**
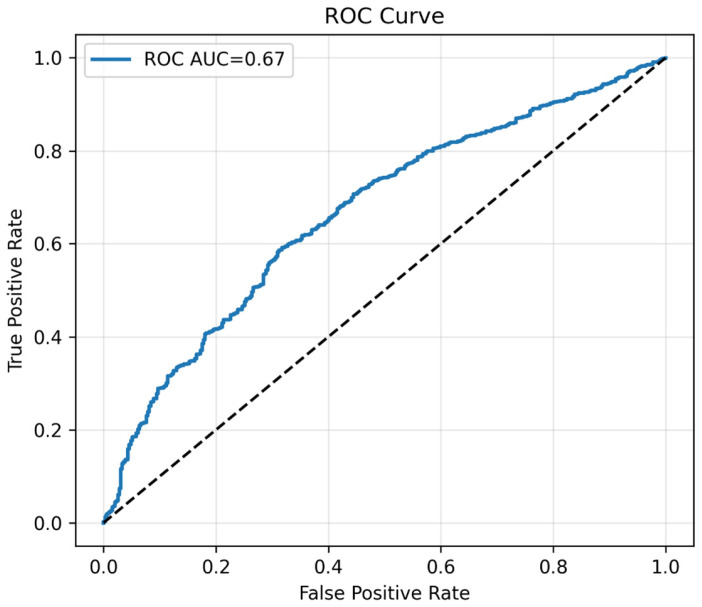
The plot of ROC curve. Note: ROC curve of the binary classifier. Demonstrates moderate discrimination, consistent with prioritization of sensitivity. The black dotted line represents the performance of a random classifier (AUC = 0.5) which indicates no significance.

The binary classification model was evaluated for its ability to distinguish between normal and prediabetes cases. Overall model performance is illustrated in the precision–recall curve (Figure 11) and the receiver operating characteristic curve (Figure 12), with corresponding metrics summarized in Table 9. The categorical model, developed using the top 20 predictors identified by permutation importance, demonstrated an overall accuracy of 0.788 and a precision of 0.791 (Table 9). Sensitivity was exceptionally high (0.995), correctly identifying almost all prediabetes cases, whereas specificity was low (0.010), indicating a tendency to classify most individuals as prediabetes. The F1 score was 0.881, reflecting a strong balance between precision and recall in this high-sensitivity context. The model achieved a PR-AUC of 0.873, highlighting good performance in ranking positive cases, while the ROC-AUC was more modest at 0.667, consistent with the class imbalance and prioritization of recall. Out of 1477 actual prediabetes cases, the model correctly classified 1473 (true positives) and missed only 4 (false negatives), but also incorrectly labeled 390 normal individuals as prediabetes (false positives). Supplementary Table S1 reports 95% bootstrap confidence intervals for ROC-AUC (0.6697–0.6882) and PR-AUC (0.6349–0.6630), quantifying the uncertainty around these estimates.

**Figure 13:**
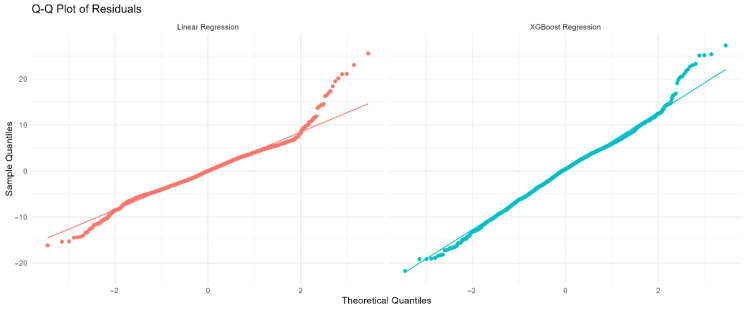
Confusion matrix at chosen threshold. Confusion matrix at the prespecified threshold of 0.50. Cell values indicate the number of true positives, false positives, true negatives, and false negatives.

At a probability threshold of 0.50, the confusion matrix (Figure 13) shows 1473 true positives, 390 false positives, 4 true negatives, and 8 false negatives.

**Figure 14:**
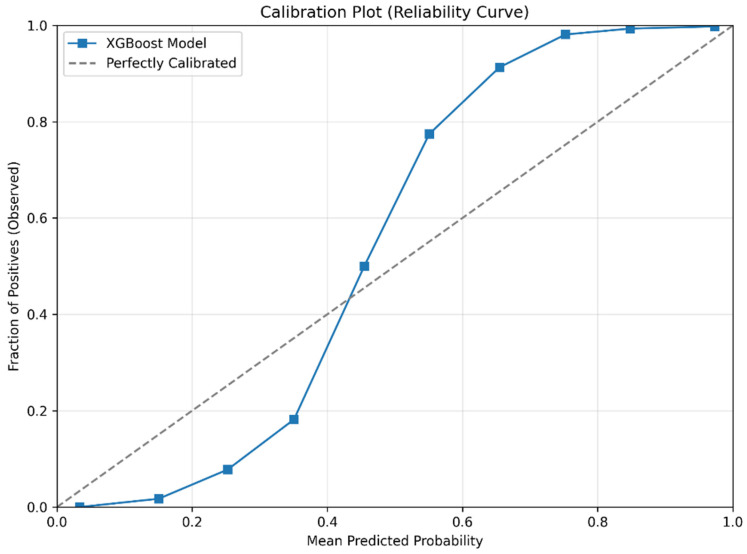
Calibration plot (reliability curve) of the XGBoost model for predicting prediabetes.

The calibration plot (Figure 14) showed predicted risks aligned well with observed outcomes across bins. It demonstrated that the model’s predicted probabilities closely aligned with observed outcomes, particularly at higher risk levels (>0.6), though slight under-prediction was observed at lower probabilities.

### 3.5. Clinical Utility and Threshold Analysis

To support clinical utility in screening settings, we evaluated a high-sensitivity operating threshold that prioritizes minimizing false negatives. At a threshold of 0.2892, the model achieved a sensitivity of 99.53% and specificity of 47.46% (Table 10). This high-sensitivity point is suitable for population-level screening, where identifying all at-risk individuals is critical, even at the cost of increased false positives.

For diagnostic settings requiring a balance between sensitivity and specificity, we identified an optimal balanced threshold using Youden’s J statistic. The threshold of 0.5683 maximized Youden’s J (0.7930), yielding a sensitivity of 88.69% and specificity of 90.61% (Table 11). This point provides a favorable trade-off between detection and precision, making it appropriate for confirmatory assessment or targeted intervention programs.

**Figure 15:**
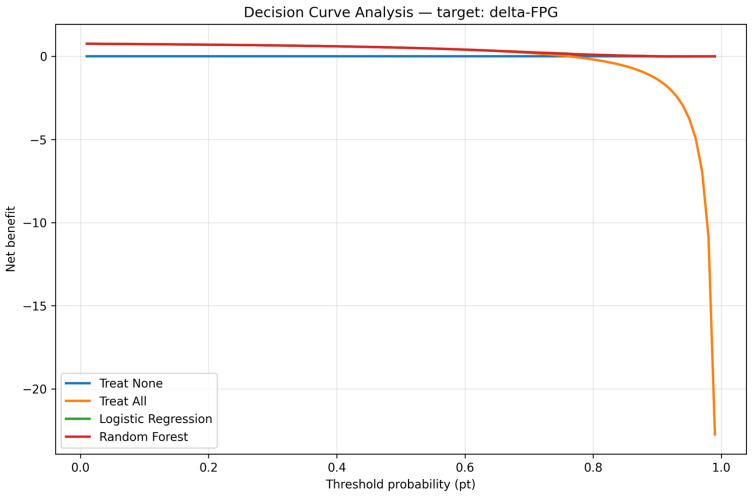
Decision curve analysis. Note: The DCA model provided superior net clinical benefit compared to ‘treat all’ and ‘treat none’.

To assess the clinical utility of our prediction model, we performed a DCA. As shown in Figure 15, the RF model provides a greater net benefit than both the ‘treat all’ and ‘treat none’ strategies across a wide range of threshold probabilities (10% to 80%). This indicates that using the model to guide screening and intervention decisions would lead to better patient outcomes than either universal screening or no screening. The model’s clinical utility is highest in the moderate risk range, supporting its use as a tool for targeted prevention in young men.

To assess probabilistic calibration, we computed the Brier score, which was 0.1754. This indicates good to moderate calibration, particularly given the inherent uncertainty in predicting long-term prediabetes risk in a relatively healthy young population. We evaluated the performance of the XGBoost model at two clinically relevant operating points. At a high-sensitivity threshold of 0.2892, the model achieved a sensitivity of 99.53% and specificity of 47.46%, minimizing false-negative classifications and making it suitable for population-level screening. In contrast, the balanced threshold (determined by Youden’s J = 0.7930) was set at 0.5683, yielding a sensitivity of 88.69% and specificity of 90.61%, representing an optimal trade-off between sensitivity and specificity for diagnostic use.

### 3.6. Model Robustness and Sensitivity Analysis

To assess the concentration of predictive signal, we removed the top three most important features from the binary XGBoost model. This reduced the ROC-AUC from 0.6753 to 0.5588 (Table 12), indicating that while the model relies on key predictors, it retains some predictive capacity even without them.

VIF values for all predictors were below 10 (max VIF = 8.00 for x8), indicating acceptable levels of multicollinearity (Supplementary Table S2). The feature correlation heatmap is shown in Supplementary Figure S2.

## 4. Discussion

In the present study, we applied four machine learning (ML) methods to identify the most important risk factors for developing prediabetes after an average follow-up of 5.9 years. FPG_base_ emerged as the dominant predictor, with an importance score of 100% in Model 1. The second most important factor, BF, reached only 17.6%. Because FPG_base_ could overshadow other variables, we constructed Model 2 by excluding it. In this model, BF, WBC, age, TSH, LDL-C, and HDL-C became the leading determinants. This dual-model strategy highlights both the overwhelming predictive power of baseline glucose and the independent contributions of metabolic, inflammatory, and lipid-related factors.

To provide further interpretability, SHAP was applied to the random forest model in Model 2. SHAP enabled not only global ranking of variable importance but also visualization of the direction and magnitude of feature effects. Although the order of features sometimes differed from that in the averaged ML rankings, this reflects methodological differences rather than inconsistency. SHAP uniquely illustrates both feature contribution and directional effect, complementing conventional importance metrics.

Beyond regression, we also developed a binary classification model to predict prediabetes status at follow-up. This approach aligns with clinical decision making, which often relies on categorical thresholds (e.g., FPG ≥ 100 mg/dL) rather than gradual changes [7]. Our classifier achieved exceptionally high sensitivity (0.995), correctly identifying nearly all prediabetes cases. While specificity was low (0.010), this trade-off suits screening programs, where minimizing false negatives is crucial. The model’s PR-AUC (0.873) confirmed strong precision–recall balance, especially important in moderately imbalanced datasets where ROC-AUC can be less informative [42].

The integration of modern ML algorithms—RF, SGB, XGBoost, and EN—into both regression and classification frameworks provides robust evidence. XGBoost and RF, in particular, consistently demonstrated high performance and stability, underscoring their suitability for structured biomedical data. Compared with deep learning, which often requires very large datasets and provides limited interpretability, these tree-based methods are efficient and clinically transparent. By combining accuracy with SHAP-based interpretability, our framework identifies modifiable risk factors that can guide preventive strategies.

Importantly, predictors overlapped across continuous and binary frameworks. BF, TG, LDL-C, and age consistently emerged as influential, underscoring their central role in early glycemic risk. The binary model further revealed threshold effects, suggesting that some predictors disproportionately influence crossing into the prediabetic range. Thus, continuous models are better suited to track metabolic trajectories, whereas binary models directly support clinical screening. Together, they provide complementary insights into early dysglycemia risk.

The strong predictive role of FPG_base_ aligns with previous longitudinal studies such as the Bogalusa Heart Study [43], which showed that childhood FPG predicted adult dysglycemia over 21 years. This reinforces FPG as a simple yet powerful marker of glucose metabolism. Similarly, BF has long been linked with impaired glucose regulation. Jo et al. [44], using NHANES data, reported higher prevalence of abnormal glucose in normal-weight individuals with elevated BF compared with overweight individuals with lower BF. Our findings further support adiposity as a central risk factor, even in young men.

Chronic inflammation, reflected by higher WBC counts, is another recognized contributor to insulin resistance [45,46]. Jiang et al. [45] found that WBC was significantly higher in Chinese individuals with prediabetes, and similar results were observed in Japanese cohorts. While the relationship between WBC and β-cell function remains debated, obesity may partly explain these conflicting findings, as it is associated with both higher WBC and altered insulin secretion.

Age also played a role, despite our cohort being restricted to young adults. Aging is associated with deterioration in glucose metabolism through mechanisms including reduced β-cell mass, oxidative stress, and impaired DNA repair [47,48,49,50,51,52,53,54]. Even subtle age effects were detectable in this population, highlighting their early contribution to glycemic risk.

TSH levels also emerged as an important factor. Prior studies have suggested associations between thyroid dysfunction, adiposity, and glucose metabolism [55,56]. Our findings add to this evidence, suggesting that thyroid function may contribute to prediabetes risk, particularly in younger individuals. Finally, dyslipidemia, reflected by elevated TG and LDL-C, was positively associated with δ-FPG. These changes are hallmarks of insulin resistance [57,58] and further underline the interdependence between lipid and glucose metabolism.

Despite these strengths, some limitations must be acknowledged. First, plasma insulin and HbA1c were not routinely available in our dataset. These biomarkers are critical for assessing insulin resistance and β-cell function, and their absence limited the comprehensiveness of our models. Second, unmeasured lifestyle factors such as detailed dietary intake, sleep quality, and family history could not be fully accounted for, potentially introducing residual confounding. Third, our cohort consisted solely of young Taiwanese men. While this enhances internal validity, it limits generalizability to women, older adults, or other ethnicities. External validation in more diverse populations is needed. Finally, although we employed repeated cross-validation and confidence interval estimation to strengthen internal validity, external or temporal validation remains a priority for future research.

## 5. Conclusions

FPG_base_ was the strongest predictor of long-term glycemic change in young Taiwanese men. Excluding FPG_base_, BF, WBC, age, TSH, LDL-C, and HDL-C remained key determinants. The dual-framework design—continuous regression and binary classification—provides complementary perspectives. Regression models capture subtle glycemic trajectories, while classification models enable threshold-based screening. Together, they enhance risk stratification and support targeted preventive strategies in young adults.

## Figures and Tables

**Table 1:** Comparison of previous studies predicting prediabetes/diabetes risk versus the present study. Includes study population, design, methods, outcomes, interpretability, and key findings, highlighting how the present dual-framework approach differs.

Study	Population	Study Design	Method(s)	Outcome	Interpretability	Key Findings	Difference from Present Study
Huang et al., 2022 [8]	Korean adults	Cross- sectional	Genetic risk score + oxidative stress score	Diabetes and prediabetes	None	Combined genetic and oxidative scores improved prediction	Not longitudinal, no ML, no SHAP
Wu et al., 2021 [9]	Middle-aged and elderly Chinese	Prospective cohort	Logistic regression	Prediabetes risk	None	Identified age, BMI, TG as key risk factors	Older population, single binary model
Yuk et al., 2022 [10]	Korean health checkup data	Cross- sectional	ML (AI algorithms)	Diabetes and prediabetes	Limited	Applied AI for screening	Cross-sectional only, no continuous δ-FPG
Liu et al., 2024 [12]	Young Chinese men (~5.8 y follow-up)	Prospective cohort	ML (RF, XGBoost, etc.)	Prediabetes	None	Developed predictive models	Did not use dual framework or SHAP
Present study (2025)	Young Taiwanese men, 18–35 y, n = 6247, mean follow-up 5.9 y	Prospective cohort	Dual ML framework (RF, SGB, XGBoost, EN vs. MLR)	Continuous δ-FPG + binary prediabetes	SHAP applied	FPG_base_ strongest predictor; BF, WBC, age, TSH, TG, LDL-C important without FPG_base_	First dual-framework ML + SHAP interpretability in young Taiwanese men; reproducibility tested with repeated runs and CI estimates

**Table 2:** Baseline demographic, biochemical, and lifestyle characteristics of the study cohort (n = 6247). Data are presented as mean ± SE for continuous variables and n (%) for categorical variables.

Variable	Mean ± SE
Age (year)	27.75 ± 5.11
Years of follow-up	5.89 ± 4.21
Body fat (%)	21.38 ± 5.50
White blood cell count (×10^3^/μL)	6.23 ± 1.44
Hemoglobin (×10^6^/μL)	15.43 ± 0.99
Platelets (×10^3^/μL)	236.72 ± 49.57
Fasting plasma glucose-baseline (mg/dL)	92.01 ± 4.74
Glutamic pyruvic transaminase (IU/L)	31.51 ± 47.78
Glutamic oxaloacetic transaminase (IU/L)	24.13 ± 20.85
γ-Glutamyl transpeptidase (IU/L)	19.88 ± 16.96
Lactate dehydrogenase (IU/L)	287.87 ± 66.74
Uric acid (mg/dL)	7.08 ± 1.40
Creatinine (mg/dL)	1.08 ± 0.13
Triglyceride (mg/dL)	100.36 ± 60.99
High-density lipoprotein cholesterol (mg/dL)	49.25 ± 11.87
Low-density lipoprotein cholesterol (mg/dL)	112.56 ± 31.15
Alkaline phosphatase (IU/L)	147.33 ± 47.38
Thyroid stimulating hormone (IU/mL)	1.61 ± 1.66
C-reactive protein (mg/dL)	0.21 ± 0.40
Drinking area	1.60 ± 7.29
Sport area	9.55 ± 9.03
δ-Fasting plasma glucose (mg/dL)	5.21 ± 7.02
Spouse status	n (%)
Single	3957 (63.9%) ***
With spouse	2232 (36.1%)
Sleep hours	n (%)
0–4 h/day	24 (0.4%)
4–6 h/day	1054 (16.9%)
6–8 h/day	4745 (76.2%)
>8 h/day	408 (6.5%)
Education level	n (%)
Primary school	3 (0.1%)
Junior high school	51 (0.8%)
Senior high school	1012 (16.2%)
College	1830 (29.3%)
University	2293 (36.8%)
Higher than master’s degree	1031 (16.8%)
Income level (thousand USD/year)	n (%)
0	1232 (19.7%) *
12.7/year	1029 (16.5%)
12.7–25.3/year	2822 (45.2%)
25.3–38.0/year	883 (14.1%)
38.0–50.6/year	130 (2.1%)
50.6–63.3/year	73 (1.2%)
>63.3/year	78 (1.2%)

*p*: * < 0.05, *** *p*: < 0.001.

**Table 3:** Equations and descriptions of performance metrics used to evaluate continuous outcome prediction models: SMAPE, RAE, RRSE, and RMSE.

Metrics	Description	Calculation
SMAPE	Symmetric mean absolute percentage error	$SMAPE=\frac{1}{n}\sum_{i=1}^{n}\frac{\left|y_{i}-{\hat{y}}_{i}\right|}{\left(\left|y_{i}\right|+\left|{\hat{y}}_{i}\right|\right)/2}\times 100$
RAE	Relative absolute error	$RAE=\sqrt{\frac{\sum_{i=1}^{n}{\left(y_{i}-{\hat{y}}_{i}\right)}^{2}}{\sum_{i=1}^{n}{\left(y_{i}-\bar{y_{i}}\right)}^{2}}}$
RRSE	Root relative squared error	$RRSE=\sqrt{\frac{\sum_{i=1}^{n}{\left(y_{i}-{\hat{y}}_{i}\right)}^{2}}{\sum_{i=1}^{n}{\left(y_{i}-\bar{y_{i}}\right)}^{2}}}$
RMSE	Root mean squared error	$RMSE=\sqrt{\frac{1}{n}\sum_{i=1}^{n}{\left(y_{i}-{\hat{y}}_{i}\right)}^{2}}$

where ${\hat{y}}_{i}$ and *y_i_* represent predicted and actual values, respectively; *n* stands the number of instances.

**Table 4:** Demographic and biochemical characteristics of participants stratified by glycemic outcome (prediabetes and normal). Continuous variables are shown as mean ± SE and categorical variables as n (%).

Variable	Prediabetes	Normal
n	2789	3458
Age (year)	28.4 ± 5.0	27.3 ± 5.1 ***
Years of follow-up	5.6 ± 4.0	6.1 ± 4.4 ***
Body fat (%)	22.1 ± 5.5	20.8 ± 5.5 ***
White blood cell count (×10^3^/μL)	6.3 ± 1.5	6.2 ± 1.4 ***
Hemoglobin (×10^6^/μL)	15.5 ± 1.0	15.4 ± 1
Platelets (×10^3^/μL)	237.1 ± 49.8	236.4 ± 49.4
Fasting plasma glucose—baseline (mg/dL)	93.3 ± 4.3	91.0 ± 4.8 ***
Glutamic pyruvic transaminase (IU/L)	32.7 ± 50.6	30.5 ± 45.4
Glutamic oxaloacetic transaminase (IU/L)	24.2 ± 20.2	24.1 ± 21.4
γ-Glutamyl transpeptidase (IU/L)	20.6 ± 17.1	19.3 ± 16.8 ***
Lactate dehydrogenase (IU/L)	290.6 ± 63	285.6 ± 69.6 ***
Uric acid (mg/dL)	7.2 ± 1.4	7.0 ± 1.4 ***
Creatinine (mg/dL)	1.1 ± 0.1	1.1 ± 0.1
Triglyceride (mg/dL)	105.8 ± 66.1	96.0 ± 56.2 ***
High-density lipoprotein cholesterol (mg/dL)	48.4 ± 11.6	50.0 ± 12.1 ***
Low-density lipoprotein cholesterol (mg/dL)	115.0 ± 32.0	110.6 ± 30.3 ***
Alkaline phosphatase (IU/L)	146.7 ± 45.0	147.8 ± 49.3
Thyroid-stimulating hormone (IU/mL)	1.6 ± 0.8	1.7 ± 2.1 *
C-reactive protein (mg/dL)	0.2 ± 0.4	0.2 ± 0.4
Drinking area	1.8 ± 7.4	1.5 ± 7.2
Sport area	9.2 ± 8.9	9.8 ± 9.1 *
δ-Fasting plasma glucose (mg/dL)	9.88 ± 5.45	1.44 ± 5.77 ***
Spouse status		
Single	1660 (60.1%)	2297 (67.1%) ***
With spouse	1104 (39.9%)	1128 (32.9%)
Sleep hours		
0–4 h/day	8 (0.3%)	16 (0.5%)
4–6 h/day	474 (17.0%)	580 (16.8%)
6–8 h/day	2129 (76.6%)	2616 (75.8%)
>8 h/day	170 (6.1%)	238 (6.9%)
Education level		
Primary school	1 (0.04%)	2 (0.1%) *
Junior high school	18 (0.7%)	33 (1.0%)
Senior high school	425 (15.3%)	587 (17.1%)
College	835 (30.0%)	995 (28.9%)
University	1006 (36.2%)	1287 (37.4%)
Higher than master’s degree	496 (17.8%)	535 (15.6%)
Income level (thousand USD/year)		
0	471 (16.9%)	761 (22.0%) ***
12.7/year	430 (15.4%)	599 (17.3%)
12.7–25.3/year	1292 (46.3%)	1530 (44.3%)
25.3–38.0/year	448 (16.1%)	435 (12.6%)
38.0–50.6/year	70 (2.5%)	60 (1.7%)
50.6–63.3/year	42 (1.5%)	31 (0.9%)
>63.3/year	36 (1.3%)	42 (1.2%)

Note: Prediabetes: subjects became prediabetic at the end of follow-up; Normal: subjects remained a normal glycemic level at the end of follow-up. *p*: * < 0.05, *** *p*: < 0.001.

**Table 5:** Average performance of linear regression and machine learning methods for predicting continuous glycemic change (δ-FPG), with and without FPG_base_.

With FPG_base_				
Methods	SMAPE	RAE	RRSE	RMSE
MLR	0.968	0.9283	0.9077	6.4156
RF	0.9571	0.9273	0.9074	6.4133
SGB	0.9426	0.9306	0.9113	6.4407
XGBoost	0.9443	0.929	0.9102	6.4329
EN	0.9599	0.9266	0.9068	6.4092
**Without** **FPG_base_**				
Methods	SMAPE	RAE	RRSE	RMSE
MLR	0.951	0.9845	0.9832	6.9022
RF	0.9477	0.9887	0.9854	6.9175
SGB	0.9489	0.9839	0.9817	6.8916
XGBoost	0.9498	0.9878	0.985	6.9149
EN	0.9492	0.9845	0.9827	6.8985

Note: MLR: multiple linear regression; RF: random forest; SGB: stochastic gradient boosting; XGBoost: eXtreme Gradient Boosting; EN: elastic net; SMAPE: symmetric mean absolute percentage error; RAE: relative absolute error; RRSE: root relative squared error; RMSE: root mean squared error.

**Table 6:** Mean and 95% confidence intervals (CIs) of performance metrics (SMAPE, RMSE, RAE, RRSE) across 10 repeated runs for continuous outcome prediction models, demonstrating reproducibility and stability.

Model	Metric	Mean	SE	95% CI Lower	95% CI Upper
EN	SMAPE	96.46007	0.016408	96.42295	96.49719
EN	RMSE	6.427868	0.00196	6.423434	6.432301
EN	RAE	0.922647	0.000325	0.921912	0.923382
EN	RRSE	0.905753	0.00021	0.905279	0.906227
RF	SMAPE	96.84203	0.021496	96.7934	96.89066
RF	RMSE	6.471158	0.00179	6.467109	6.475208
RF	RAE	0.920818	0.000309	0.920119	0.921517
RF	RRSE	0.912466	0.000341	0.911695	0.913237
XGBoost	SMAPE	104.8296	0.024476	104.7742	104.8849
XGBoost	RMSE	7.056346	0.002225	7.051313	7.06138
XGBoost	RAE	1.004276	0.000322	1.003548	1.005003
XGBoost	RRSE	0.994844	0.000363	0.994023	0.995666

Note: SMAPE: symmetric mean absolute percentage error; RMSE: root mean squared error; RAE: relative absolute error; RRSE: root relative squared error. RF: random forest; XGBoost: eXtreme Gradient Boosting, EN: elastic net.

**Table 7:** Relative importance percentages of predictors in Model 1 (with FPG_base_ included) across four machine learning methods (RF, SGB, XGBoost, EN).

Variables	MOIP
Baseline fasting plasma glucose	100
Body fat	17.64
Thyroid-stimulating hormone	14.8
White blood cell count	14.51
Years of follow-up	14.32
C-reactive protein	11.63
Age	10.30
HDL-cholesterol (HDL-C)	9.75
Uric acid	9.34
Hemoglobin	8.80
Lactic dehydrogenase	8.32
LDL-cholesterol (LDL-C)	8.30
Triglyceride (TG)	7.63
Alkaline phosphatase	7.39
Platelet	7.11
Glutamic pyruvic transaminase (GPT)	5.93
γ-Glutamyl transpeptidase	5.27
Spouse status	4.87
Glutamic oxaloacetic transaminase (GOT)	4.50
Sport area	4.27
Income status	3.50
Creatinine	2.41
Sleeping level	2.15
Education level	1.71
Drinking area	0.54

Note: RF: random forest; SGB: stochastic gradient boosting; XGBoost: eXtreme Gradient Boosting; EN: elastic net; MOIP: mean of importance percentage across four ML models (RF, SGB, XGBoost, EN). Body fat: adiposity marker—consistently pro-glycemic. White blood cell count: inflammatory marker—higher levels indicate chronic low-grade inflammation linked to insulin resistance. Thyroid-stimulating hormone: thyroid function marker—higher levels associated with reduced metabolic rate and increased adiposity. TG, LDL-C, HDL-C: lipid panel—core components of insulin resistance dyslipidemia (high TG, high LDL-C, low HDL-C). Age: aging marker —associated with declining β-cell function and increasing insulin resistance.

**Table 8:** Relative importance percentages of predictors in Model 2 (excluding FPG_base_) across four machine learning methods (RF, SGB, XGBoost, EN).

Variables	MOIP
Body fat	88.85
White blood cell count	58.62
Age	54.89
Thyroid stimulating hormone	36.87
Triglyceride	32.66
LDL-cholesterol	28.69
HDL-cholesterol	27.42
Years of follow-up	24.94
γ-Glutamyl transpeptidase	22.78
Hemoglobin	22.67
Lactic dehydrogenase	22.32
Alkaline phosphatase	22.16
Platelet	21.36
Uric acid	20.95
Glutamic oxaloacetic transaminase (GOT)	18.88
Glutamic pyruvic transaminase (GPT)	18.49
Sport area	16.83
Creatinine	8.95
Income level	6.71
Education level	6.11
C-reactive protein	5.14
Sleeping level	4.00
Drink area	1.65
Spouse status	0

Note: RF: random forest; SGB: stochastic gradient boosting; XGBoost: eXtreme Gradient Boosting; EN: elastic net; MOIP: mean of importance percentage across four ML models (RF, SGB, XGBoost, EN). Body fat: adiposity marker—consistently pro-glycemic. White blood cell count: inflammatory marker—higher levels indicate chronic low-grade inflammation linked to insulin resistance. Thyroid-stimulating hormone: thyroid function marker—higher levels associated with reduced metabolic rate and increased adiposity. TG, LDL-C, HDL-C: lipid panel—core components of insulin resistance dyslipidemia (high TG, high LDL-C, low HDL-C). Age: aging marker—associated with declining β-cell function and increasing insulin resistance.

**Table 9:** Performance metrics of the categorical (binary) model for predicting prediabetes versus normal outcomes, including accuracy, precision, recall, specificity, false positive rate, F1 score, ROC-AUC, PR-AUC, and classification counts.

Accuracy	Precision	Sensitivity (Recall)	Specificity	False Positive Rate	F1 Score	ROC-AUC	PR-AUC	True Positives	False Positives	True Negatives	False Negatives
0.788	0.791	0.995	0.010	0.990	0.881	0.667	0.873	1473	390	4	8

**Table 10:** Performance of the binary model at a high-sensitivity threshold (0.2892), prioritizing minimization of false negatives for screening purposes.

Metric	Value
Threshold	0.2892
Sensitivity	0.9953
Specificity	0.4746

**Table 11:** Performance of the binary model at the optimal balanced threshold (0.5683), determined by Youden’s J statistic, representing a trade-off between sensitivity and specificity suitable for diagnostic settings.

Metric	Value
Threshold	0.5683
Sensitivity	0.8869
Specificity	0.9061
Youden’s J	0.7930

**Table 12:** Sensitivity and ablation analysis.

Model	ROC-AUC
Original XGBoost	0.6753
XGBoost (top 3 features removed)	0.5588

## Data Availability

Data available on request due to privacy/ethical restrictions. A minimal, anonymized analysis code template is available from the corresponding author upon reasonable request.

## Supplementary Materials

The following supporting information can be downloaded at https://www.mdpi.com/article/10.3390/diagnostics15192507/s1. Figure S1: Feature correlation heatmap; Figure S2: Simplified version of our modeling pipeline; Table S1: Classification performance with 95% bootstrap confidence intervals for ROC_AUC and PR_AUC; Table S2: feature_vif_table.csv.
