# Supplementary material for: Dual Machine Learning Framework for Predicting Long-Term Glycemic Change and Prediabetes Risk in Young Taiwanese Men
Source: Diagnostics (Basel). 2025 Oct 2;15(19):2507. doi: 10.3390/diagnostics15192507 (PMC12524205; doi:10.3390/diagnostics15192507)
Supplement: Supplementary file 1 [file diagnostics-15-02507-s001.zip › diagnostics-3836960-supplementary.pdf]

Supplementary Table S1. Classification performance with 95% bootstrap confidence intervals for ROC_AUC and PR_AUC

Model	ROC_AUC_95CI_ Lower	ROC_AUC_95CI_ Upper	PR_AUC_95CI_ Lower	PR_AUC_95CI_ Upper
XGBoost Classification	0.6697	0.6882	0.6349	0.6630

Confidence intervals were estimated using 1000 bootstrap resamples. Point estimates for accuracy, precision, recall, and F1-score are reported in Table 9

Supplementary Table S2

feature_vif_table.csv

Feature	VIF
x8	8.001215
x7	7.999843
x1	3.142243
x20	2.209107
x19	1.688071
x13	1.494205
x2	1.484577
x10	1.468823
x9	1.445064
x6	1.297391
x14	1.233156
x24	1.224945
x12	1.224187
y	1.21885
x15	1.196231
x3	1.19485
x5	1.160686
x11	1.108257
x18	1.102081
x4	1.091482
x25	1.080169
x22	1.059056
x17	1.056777

x21	1.048533
x23	1.025604
x16	1.012438

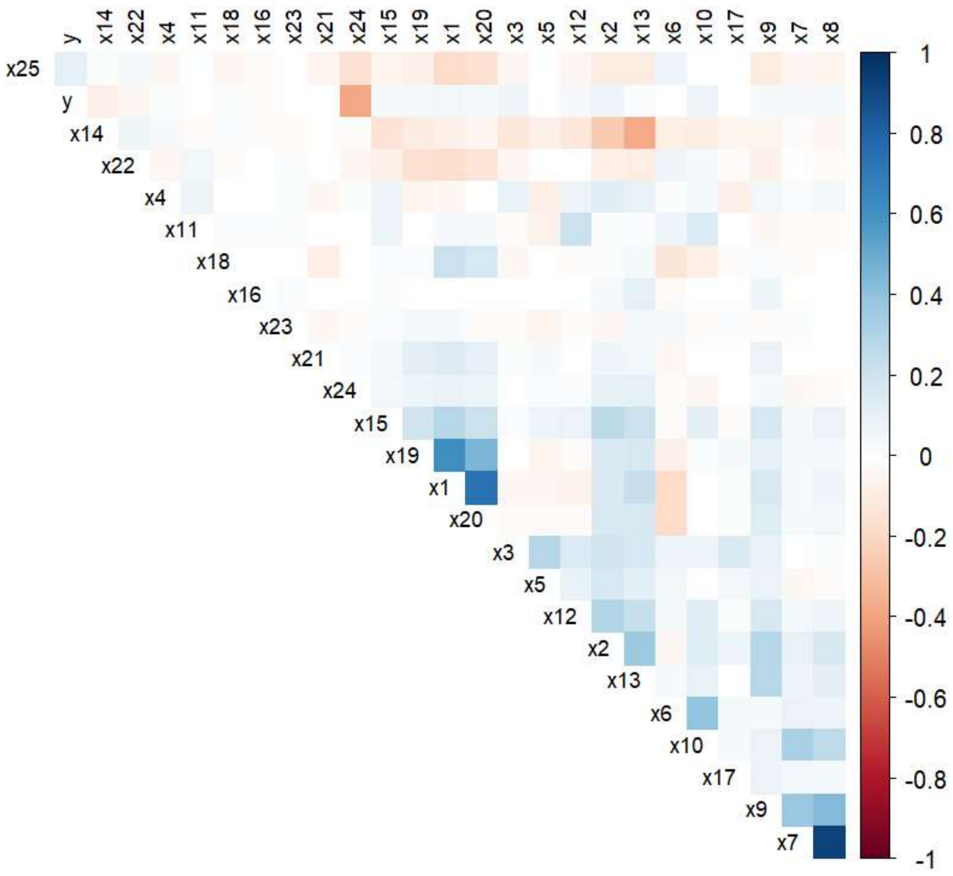

Supplementary Figure S1 Feature correlation heatmap

Strong red/blue squares (e.g., GOT–GPT, LDL-C–Total Chol) indicate potential collinearity — interpret SHAP importance cautiously for these pairs.

```
import pandas as pd
import numpy as np
import xgboost as xgb
import shap

# Generate synthetic data mimicking your cohort
np.random.seed(42)
n = 100
data = pd.DataFrame({
    'Age': np.random.randint(18, 36, n),
    'BodyFat': np.random.normal(21, 5, n),
    'WBC': np.random.normal(6.2, 1.4, n),
    'TSH': np.random.exponential(1.6, n),
    'TG': np.random.gamma(2, 50, n),
    'LDL_C': np.random.normal(112, 31, n)
})
data['delta_FPG'] = (
    0.1 * data['Age'] +
    0.05 * data['BodyFat'] +
    np.random.normal(0, 2, n)
)

# Train XGBoost
X, y = data.drop('delta_FPG', axis=1), data['delta_FPG']
model = xgb.XGBRegressor(n_estimators=10, max_depth=3, random_state=42)
model.fit(X, y)

# SHAP
explainer = shap.Explainer(model)
shap_values = explainer(X)

# Output a metric
rmse = np.sqrt(np.mean((model.predict(X) - y) ** 2))
print(f"Synthetic RMSE: {rmse:.3f}")
```

Supplementary Figure S2 Simplified version of our modeling pipeline
